# Supplementary material for: The value of long-duration energy storage under various grid conditions in a zero-emissions future
Source: Nat Commun. 2024 Nov 3;15:9501. doi: 10.1038/s41467-024-53274-6 (PMC11532502; doi:10.1038/s41467-024-53274-6)
Supplement: Supplementary file 1 — Supplementary Information [file 41467_2024_53274_MOESM1_ESM.pdf]

The Value of Long-Duration Energy Storage under Various Grid Conditions in a Zero-Emissions Future

Supplemental Information

Martin Staadecker,[1,2,*] Julia Szinai,[3] Pedro A. Sánchez-Pérez,[4] Sarah Kurtz,[5] and Patricia Hidalgo-Gonzalez[1,6,*]

[1]Mechanical and Aerospace Engineering, University of California San Diego, La Jolla, CA 92093, USA
[2]Division of Engineering Science, University of Toronto, Toronto, ON M5S 1A1, Canada
[3]Lawrence Berkeley National Laboratory, Berkeley, CA 94720, USA
[4]National Renewable Energy Laboratory, Golden, CO 80401, USA
[5]School of Engineering, University of California Merced, Merced, CA 95340, USA
[6]Center for Energy Research, University of California San Diego, La Jolla, CA 92093, USA
*Corresponding authors: martin.staadecker@mail.utoronto.ca and phidalgogonzalez@ucsd.edu

Contents

Supplemental Tables 3

Supplementary Table 1. Storage Characteristics in California under Varying Energy Storage Costs 3

Supplementary Table 2. Existing and Available Capacities by Technology Used in the Model 3

Supplementary Table 3. Comparison of baseline 4-hour temporal resolution scenario to 1-hour temporal resolution sensitivity scenario 4

Supplementary Table 4. Technology assumptions for existing and candidate generators. 5

Supplementary Table 5. Average capital, fixed O&M, and variable O&M costs for candidate generation 5

Supplementary Figures 7

Supplementary Figure 1. Load zones and transmission lines used by the SWITCH model. 7

Supplementary Figure 2. Impact of Four Factors on the Use and Value of LDES Using Net Amounts 8

Supplementary Figure 3. Regional Impacts of 50% Hydropower Reduction on Storage, Generation, and Transmission 9

Supplementary Figure 4. Impacts on Storage of 10x Increase in Transmission Expansion Costs 9

Supplementary Figure 5. Seasonal Cycles in the Energy Stored in the WECC under Varying Storage Cost Scenarios 10

Supplementary Figure 6. Daily dispatch curve, comparison of baseline to 1-hour resolution sensitivity 10

Supplementary Note 1. 364 Day Model 11

Supplementary Note 2. SWITCH Mathematical Formulation 11

Supplementary Methods 12

Financial Data 12

Load Zones 12

Existing Generators 12

Candidate Generators 13

Technology assumptions on lifetime, capacity factors, efficiencies 14

Transmission 14

Overnight capital cost, variable O&M cost, fixed O&M cost 14

Connection costs 14

Fuel costs and Emissions 15

Transmission costs 15

Load 15

Carbon cap 15

Supplemental References 17

Supplemental Tables

Supplementary Table 1. Storage Characteristics in California under Varying Energy Storage Costs

Energy Storage Cost ($/kWh)	California energy storage capacity (TWh)	California median storage duration (hours)	California largest storage duration (hours)
102	0.70	6.3	7.9
22 (Baseline)	0.74	7.1	8.2
10	0.80	7.3	11
5	3.4	19	212
2	9.7	134	474
1	10	159	620
0.5	16	163	787

For all scenarios, storage power capital costs are 19.58$/kW while operation and maintenance costs are 6.10$/kW-year. The baseline costs represent a scenario where the Department of Energy's "Energy Storage Grand Challenge" is achieved (90% reduction in storage costs by 2030).

$ = 2018 US dollars, kWh = kilowatt-hours, TWh = terawatt-hours, kW = kilowatt.

Supplementary Table 2. Existing and Available Capacities by Technology Used in the Model

Energy Source	Generation Technology	Total Existing Capacity (GW)	Existing Capacity Still Online in 2050 (GW)	Maximum Available Capacity (GW)
**Biogas**	Biogas	0.2	0.0	No max. Constrained by fuel costs
	Biogas Internal Combustion Engine	0.4	0.0	0.0
	Biogas Internal Combustion Engine Cogen	0.2	0.0	0.1
	Biogas Steam Turbine	0.1	0.0	0.0
**Bio Liquid**	Bio Liquid Steam Turbine Cogen	0.5	0.0	0.3
**Bio Solid**	Bio Solid Steam Turbine	0.7	0.0	0.0
	Bio Solid Steam Turbine Cogen	0.6	0.1	0.4
**Coal**	IGCCC	0.0	0.0	Effectively 0.0 due to zero-emissions constraint
	Coal Steam Turbine	95.4	3.3	Effectively 0.0 due to zero-emissions constraint
	Coal Steam Turbine Cogen	0.5	0.0	Effectively 0.0 due to zero-emissions constraint
**Distillate Fuel Oil**	Distillate Fuel Oil Combustion Turbine	0.5	0.0	0.0
	Distillate Fuel Oil Internal Combustion Engine	0.9	0.0	0.0
**Electricity**	Battery Storage	1.1	0.0	0.0
	LDES	0.0	0.0	No max. See main paper for details.
**Gas**	CCGT	67.1	8.9	Effectively 0.0 due to zero-emissions constraint
	CCGT Cogen	7.7	0.0	Effectively 0.0 due to zero-emissions constraint
	Gas Combustion Turbine	31.1	6.9	Effectively 0.0 due to zero-emissions constraint
	Gas Combustion Turbine Cogen	4.5	0.0	Effectively 0.0 due to zero-emissions constraint

	Gas Internal Combustion Engine	1.7	0.0	0.0
	Gas Internal Combustion Engine Cogen	0.5	0.0	Effectively 0.0 due to zero-emissions constraint
	Gas Steam Turbine	38.1	0.2	0.0
	Gas Steam Turbine Cogen	0.4	0.0	Effectively 0.0 due to zero-emissions constraint
	Other Turbine	0.0	0.0	0.0
**Geothermal**	Geothermal	13.3	0.0	11.1
**Residual Fuel Oil**	Gas Combustion Turbine	0.2	0.0	0.0
**Solar**	Central PV	26.6	0.0	3270.2
	Commercial PV	0.0	0.0	52.7
	Residential PV	0.0	0.0	125.3
	Concentrating Solar Power Trough, No Storage	0.4	0.0	5362.5
	Concentrating Solar Power Trough, 6h Storage	0.0	0.0	3695.3
	Steam Turbine	1.6	0.0	0.0
**Uranium**	Nuclear	14.3	9.6	0.0
**Waste Heat**	Other Turbine	0.0	0.0	0.0
	Steam Turbine	0.2	0.0	0.0
**Water**	Hydropower	239.3	239.3	0.0
	Pumped Storage	12.4	12.4	0.0
**Wind**	Onshore Wind	42.3	0	521.4
	Offshore Wind	0.0	0.0	6.4
**Total**		**602.8**	**280.7**	**N/A**
CCGT = combined cycle gas turbine, IGCCC = integrated gasification combined cycle, coal. GW = gigawatts, LDES = long-duration Energy Storage, PV = photovoltaics.				

Supplementary Table 3. Comparison of baseline 4-hour temporal resolution scenario to 1-hour temporal resolution sensitivity scenario

Value to compare	Baseline (4-hour)	Sensitivity (1-hour)	Difference between sensitivity and baseline
Yearly demand (TWh)	1,458	1,469	11 (+0.8%)
Mean load (GW)	166.9	168.2	1.3 (+0.8%)
Total generation and storage capacity (GW)	849.1	890.7	41.6 (+4.9%)
Mean Storage duration (hours)	7.05	7.18	0.13 (+1.8%)
Storage energy capacity (TWh)	1.938	1.993	0.055 (+2.8%)
Storage power capacity (GW)	237.5	255.7	18.2 (+7.6%)
Solar capacity (GW)	430.2	441.8	11.6 (+2.7%)
Wind capacity (GW)	98.9	109.0	10.1 (+10.2%)
Biomass capacity (GW)	9.2	10.9	1.7 (+18.7%)
Geothermal capacity (GW)	0.0	0.0	No change
Hydropower (GW)	66.3	66.3	No change

Transmission capacity (million MW-km)	122.9	131.1	8.2 (+6.6%)
Mean daily curtailment (GWh)	321.5	300.1	-21.4 (-6.7%)
Values are aggregates for the Western Interconnect.<br>TWh = terawatt-hours, GW = gigawatts, MW-km = megawatt-kilometers, GWh = gigawatt-hours.			

Supplementary Table 4. Technology assumptions for existing and candidate generators.

Fuel Source	Generating Technology	Lifetime (Years)	Forced Outage Rate (%)	Scheduled Outage Rate (%)
**Biogas**	Biogas	20	4%	6%
	Biogas Internal Combustion Engine	20	4%	6%
	Biogas Internal Combustion Engine Cogen	20	11%	4%
	Biogas Steam Turbine	20	13%	9%
**Bio Liquid**	Bio Liquid Steam Turbine Cogen	40	13%	9%
**Bio Solid**	Bio Solid Steam Turbine	20	4%	6%
	Bio Solid Steam Turbine Cogen	40	13%	9%
**Electricity**	Battery Storage	10	2%	1%
	LDES	15	2%	0.55%
**Geothermal**	Geothermal	20	0%	0%
**Solar**	Central PV	20	0%	0%
	Commercial PV	20	0%	0%
	Concentrating Solar Power Trough 6h Storage	20	6%	0%
	Concentrating Solar Power Trough No Storage	20	0%	0%
	Residential PV	20	0%	0%
	Steam Turbine	20	4%	6%
**Uranium**	Nuclear	80	4%	6%
**Waste Heat**	Other Turbine	40	4%	6%
	Steam Turbine	40	4%	6%
**Water**	Hydropower	200	5%	5%
	Pumped Storage	200	5%	5%
**Wind**	Offshore Wind	30	5%	1%
	Onshore Wind	20	0%	0%
Note: Values for emitting generators (e.g., coal, natural gas, etc.) are not shown since these generators do not contribute to the model dispatch due to the zero-emissions constraint.<br>PV = photovoltaic, LDES = long-duration energy storage.				

Supplementary Table 5. Average capital, fixed O&M, and variable O&M costs for candidate generation

Energy Source	Generation Technology	Overnight capital cost ($/MW)	Fixed O&M cost ($/MW-year)	Variable O&M cost ($/MWh)
Bio Gas	Bio Gas	2,118,354	64,380	9.20
	Bio Gas Internal Combustion Engine Cogen	1,588,766	48,285	16.00
Bio Liquid	Bio Liquid Steam Turbine Cogen	3,225,737	80,012	16.80
Bio Solid	Bio Solid Steam Turbine Cogen	3,225,737	80,012	16.80
Electricity	LDES	19,580	6,096	0.0
Geothermal	Geothermal	6,970,164	173,105	0.0
Solar	Central PV	702,651	8,229	0.0
	Commercial PV	819,995	5,879	0.0

	CSP Trough 6h Storage	4,023,815	52,447	3.59
	CSP Trough No Storage	3,937,151	56,149	0.0
	Residential PV	884,353	6,633	0.0
Wind	Offshore Wind	2,226,776	112,298	0.0
	Onshore Wind	1,042,433	33,692	0.0
Note: Costs for emitting generators (e.g., coal, natural gas, etc.) are not shown since these generators do not contribute to the model dispatch due to the zero-emissions constraint.<br><br>CSP = concentrated solar power, PV = photovoltaic, LDES = long-duration energy storage, $ = 2018 US dollars, MW = megawatts, MWh = megawatt-hours.				

Supplementary Figures

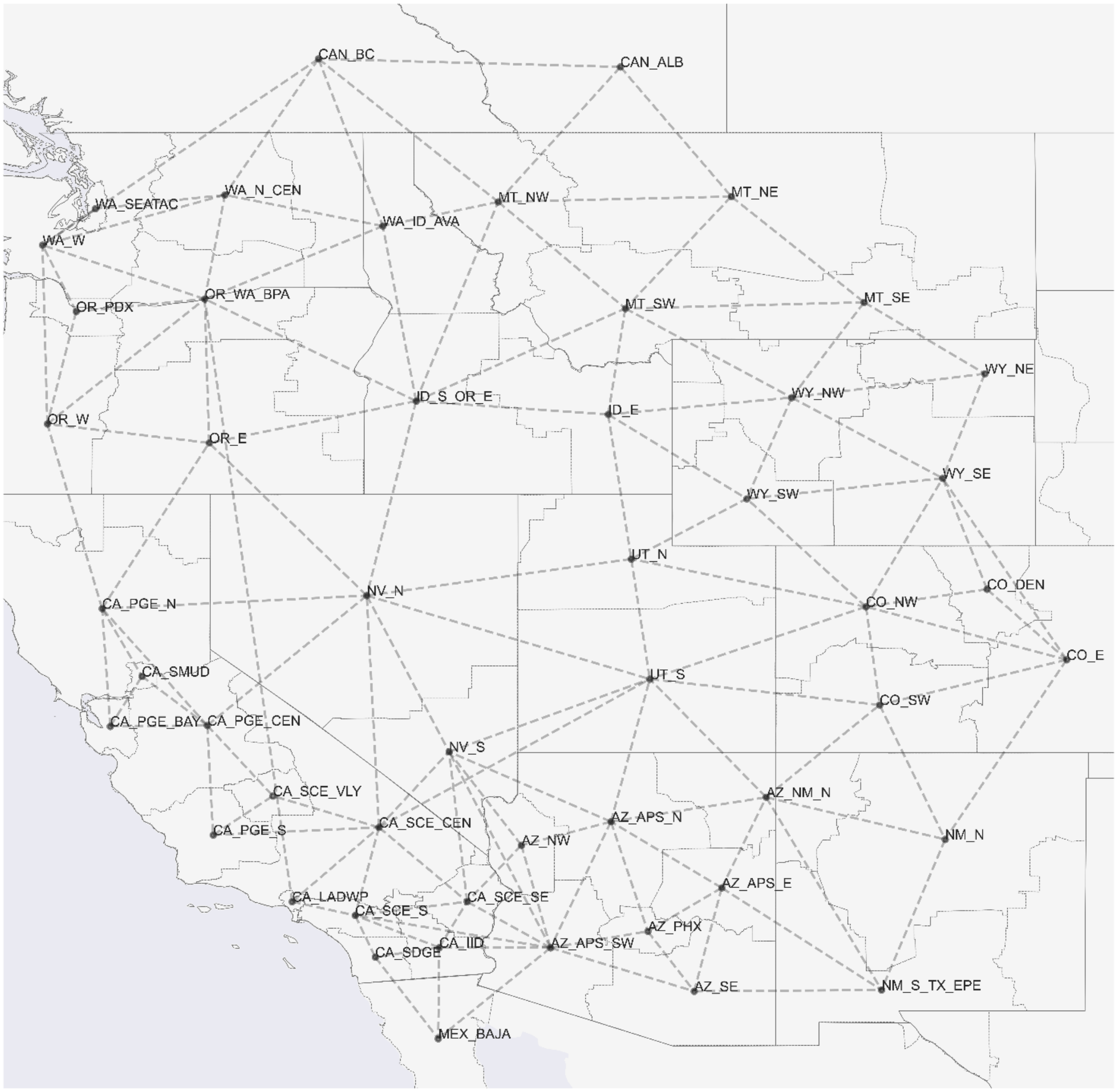

Supplementary Figure 1. Load zones and transmission lines used by the SWITCH model.

The load zone and transmission lines used by the SWITCH model. Transmission lines represent every possible transmission corridor where transmission capacity may already exist and where new transmission capacity may be built. The labels (e.g., CA_SDGE) display each load zone's unique identifier.

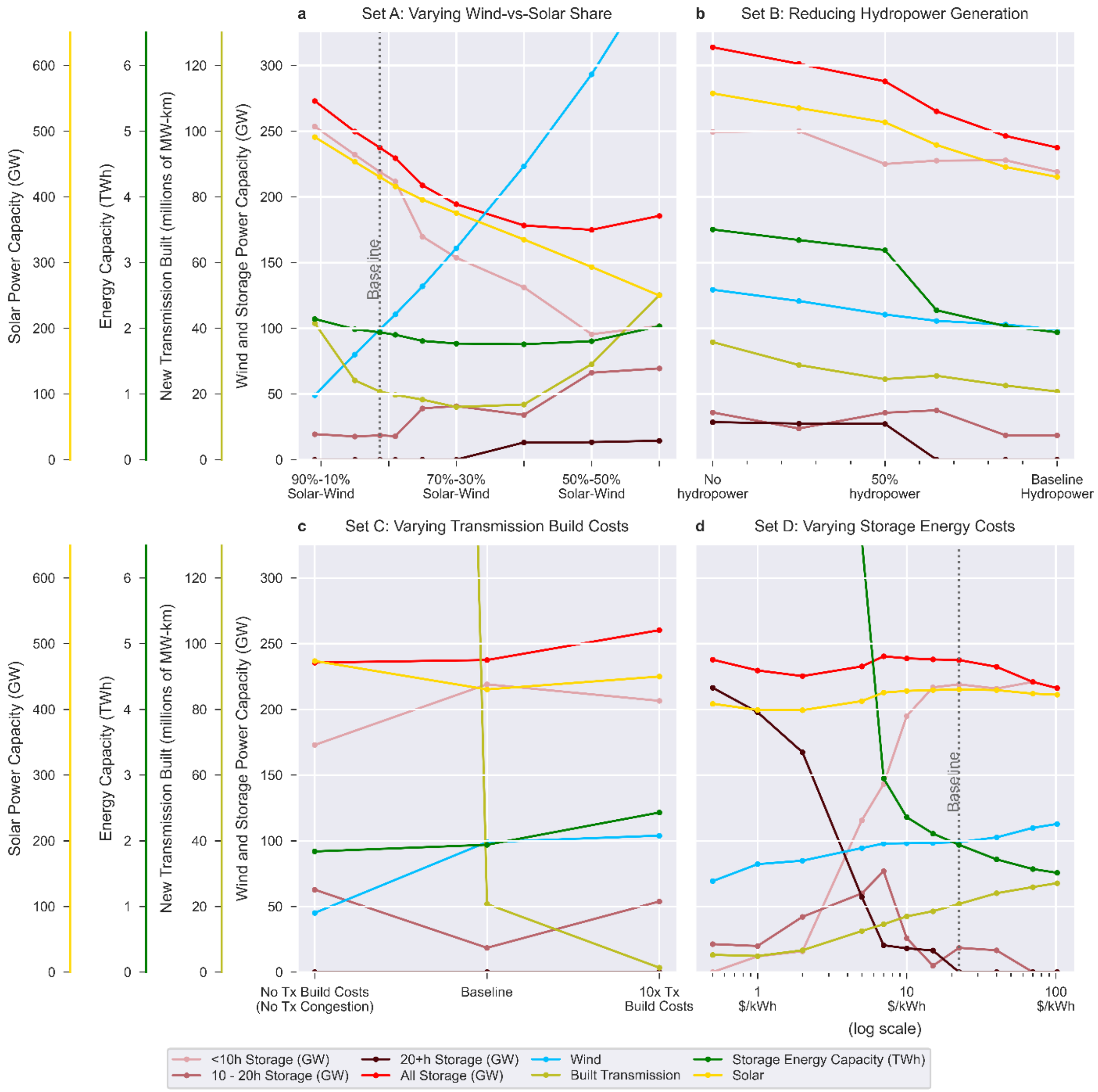

Supplementary Figure 2. Impact of Four Factors on the Use and Value of LDES Using Net Amounts

Highlights the change in storage duration, built capacity and transmission capacity as one of 4 factors vary (panels a to d). Equivalent to Fig. 2 in the main text except that values are net amounts rather than percentages changed relative to the baseline.

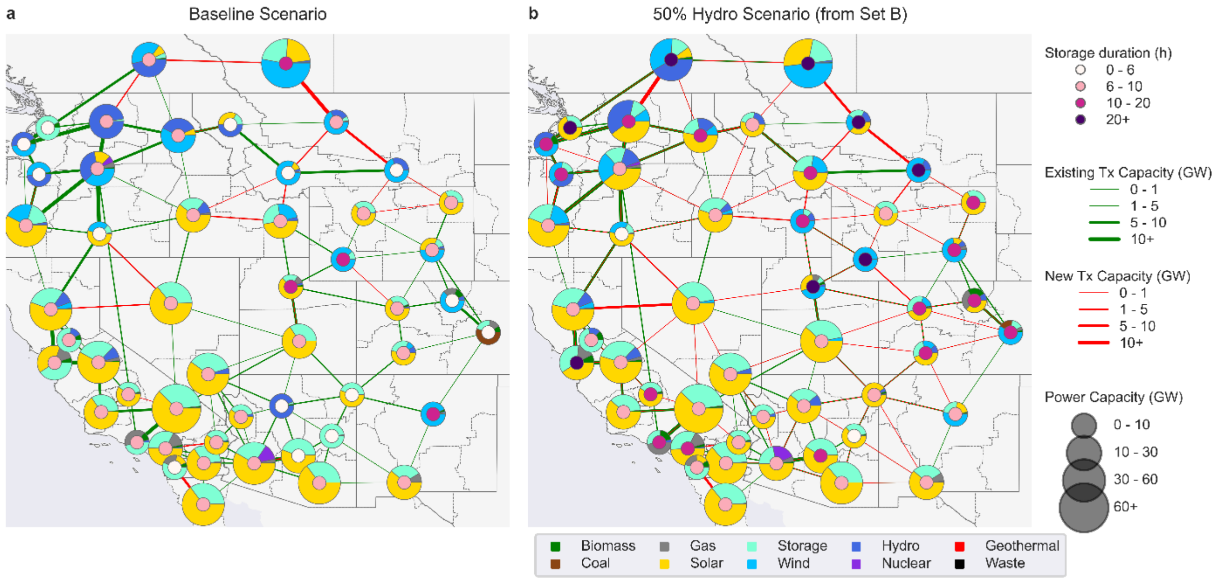

Supplementary Figure 3. Regional Impacts of 50% Hydropower Reduction on Storage, Generation, and Transmission

Spatial comparison of the baseline (a) to the 50% hydropower reduction scenario (b). Regions that previously relied on hydropower show an increase in the average duration of storage.

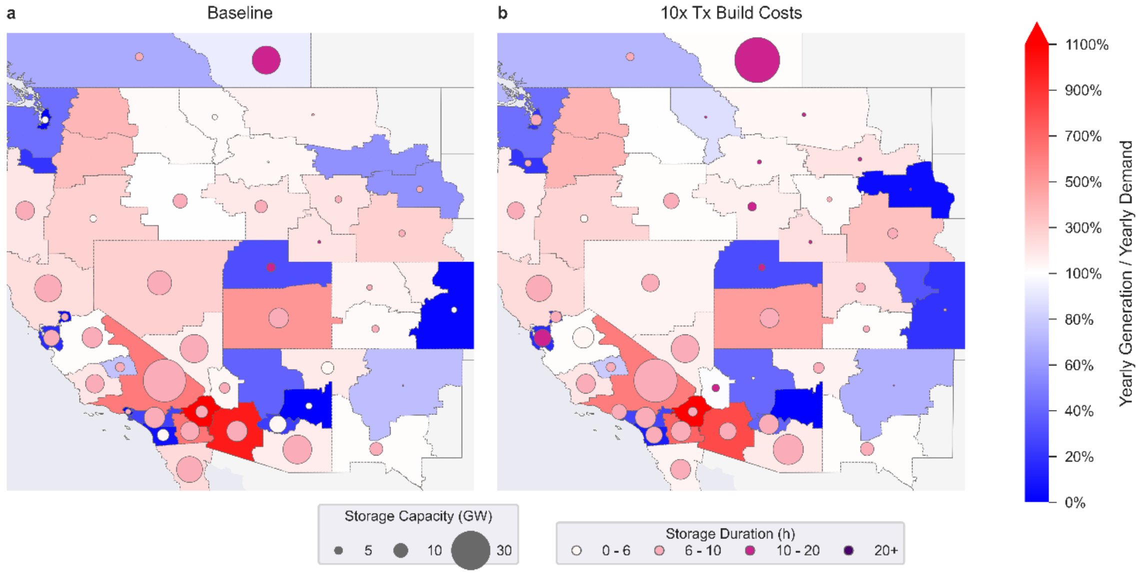

Supplementary Figure 4. Impacts on Storage of 10x Increase in Transmission Expansion Costs

Comparison of the baseline (a) with the 10x increased transmission (tx) expansion cost scenario (b). Storage is only impacted in a handful of transmission-dependent load zones (e.g. Alberta). Blue load zones generate less than their yearly demand (net importers), and red load zones generate more than their yearly demand (net exporters). Circle size represents storage power capacity. Circle color represents storage duration.

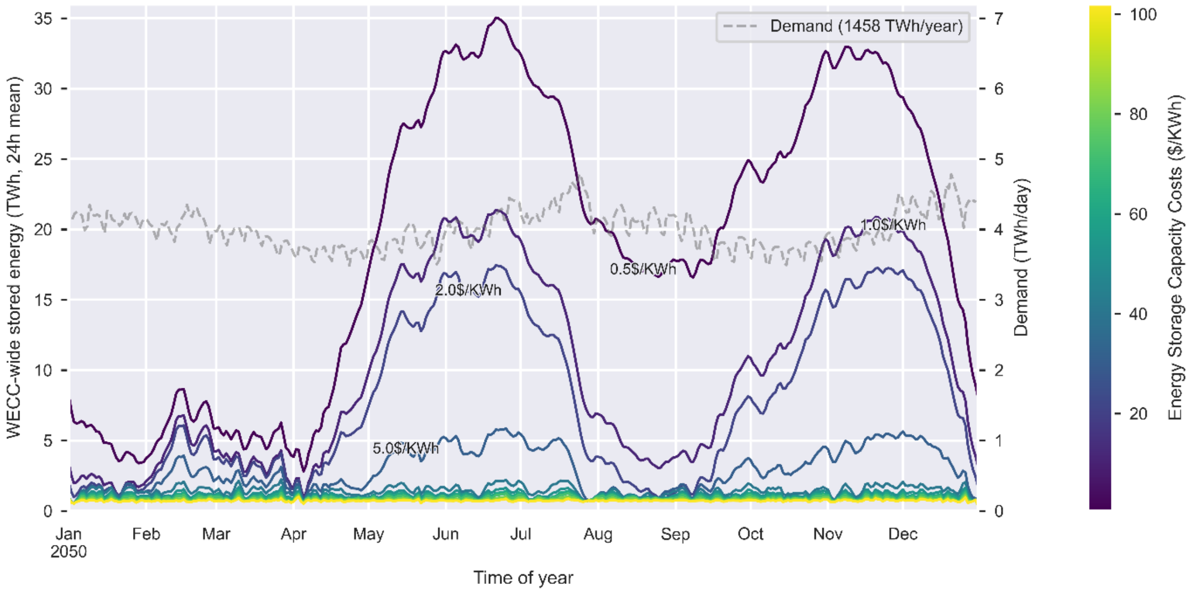

Supplementary Figure 5. Seasonal Cycles in the Energy Stored in the WECC under Varying Storage Cost Scenarios

Depiction of the seasonal change in the energy held in storage (“state of charge”). Storage resources are operated on seasonal cycles when energy storage capacity costs are at or below 5 $/kWh. Yearly cycles emerge when costs are at or below 1 $/kWh.

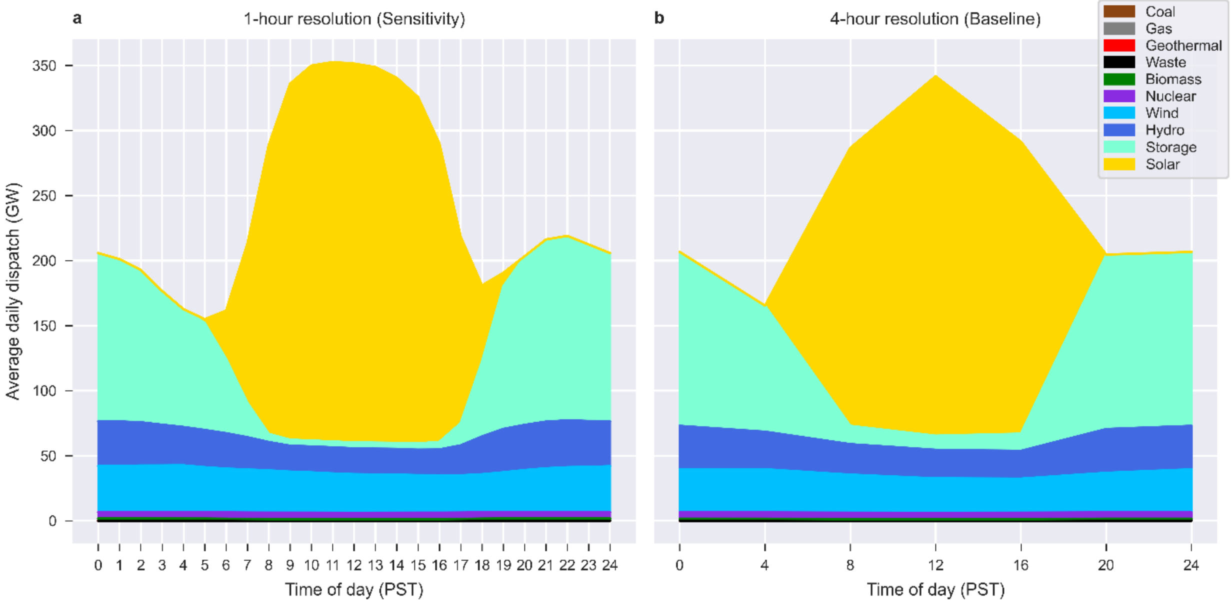

Supplementary Figure 6. Daily dispatch curve, comparison of baseline to 1-hour resolution sensitivity

Comparison of the baseline’s average daily dispatch curve (b) against a model with hourly temporal resolution (a).

Supplementary Note 1. 364 Day Model

No data was used for January 1st since data was missing due to time zone conversions. Therefore, the model is a 364 day model not a 365 day model. The term 365 day (or similar terms like "every day of the year") is not technically correct but best describes in simple terms the resolution of our model (i.e.: comparable to other 365-day models). Hence, we still use the term 365 day (or similar) in the paper.

Supplementary Note 2. SWITCH Mathematical Formulation

The model used in this paper has the same mathematical formulation as the original SWITCH 2.0 model described in the Supplementary Materials of Johnston et al.[1] except for the following changes.

We only used the following modules from SWITCH 2.0:

- switch_model
- switch_model.timescales
- switch_model.financials
- switch_model.balancing.load_zones
- switch_model.energy_sources.properties
- switch_model.generators.core.build
- switch_model.generators.core.dispatch
- switch_model.reporting
- switch_model.generators.core.no_commit
- switch_model.generators.extensions.hydro_simple
- switch_model.generators.extensions.storage
- switch_model.energy_sources.fuel_costs.markets
- switch_model.transmission.transport.build
- switch_model.transmission.transport.dispatch
- switch_model.policies.carbon_policies

The storage module (switch_model.generators.extensions.storage) was modified to allow for separate charging and discharging efficiency parameters ($\eta_g^{S,in}$ and $\eta_g^{S,out}$) instead of a single round-trip efficiency parameter. Specifically, equation (60) of the SWITCH 2.0 supplementary materials[1] was changed to supplementary equation (1).

$$Z_{g,t}^S = Z_{g,t-1}^S + \left(\eta_g^{S,in} C_{g,t}^S - \frac{1}{\eta_g^{S,out}} P_{g,t}\right) \Delta_t^T \qquad \forall g \in G^S, t \in T_g^{on} \tag{1}$$

A custom module (switch_model.policies.min_per_tech) was added to the model to support energy capacity mandates. The module accepts a parameter $min_{tech,p}^{energy}$ which represents the minimum amount of energy capacity (in MWh) needed throughout the model for a given storage technology and period. The module adds supplementary equation (2) as a constraint to the model.

$$\sum_{g \in G_{tech}^T \cap G^S} K_{g,p}^S \geq min_{tech,p}^{energy} \qquad \forall p \in P, tech \in TECHS \tag{2}$$

A custom module (switch_model.policies.wind_to_solar_ratio) was added to the model. This module accepts a parameter ($ratio_p$) that is used in the supplementary equation (3) constraint to fix the ratio of wind to solar capacity.

$$\sum_{g \in G_{tech1}^T} K_{g,p}^S = ratio_p * \sum_{g \in G_{tech2}^T} K_{g,p}^S, \quad \text{where tech1=Wind, tech2=Solar} \tag{3}$$

We create a new "hydro timeseries" which allows for hydropower generation to correspond to the average monthly capacity factors as previously described.

Other minor changes were made as described here: https://github.com/REAM-lab/switch/releases/tag/v2.0.0

Supplementary Methods

This section describes the data sources used for the SWITCH model.

Financial Data

5% was used for the interest rate and discount rate in the model to reflect the general decrease in US interest rates in recent years. All costs in the model are 2018 costs.

Load Zones

The SWITCH model divides the WECC into 50 “load zone” regions (supplementary figure 5). Load zone boundaries were used from prior SWITCH-WECC analyses,[2,3] which constructed the regions based on state lines, North American Electric Reliability Corporation (NERC) control areas, utility service territory boundaries, and high-population metropolitan areas. Some utility service territories were divided into multiple zones if there was a large amount of high-voltage transmission connectivity existing within the same service territory (i.e. CA PGE BAY, CA PGE CEN, CA PGE S, CA PGE N for PG&E). The boundaries were also constructed to represent zones that are unlikely to have transmission congestion within the zone (since only transmission between zones is included), and between which there has historically been transmission congestion (reflecting pathways where additional transmission may be needed).

Existing Generators

As inputs into SWITCH, we include the list of individual generators that are existing and/or are planned for the WECC region. For the US portion of WECC, we extract the list of generators and their characteristics (such as location, fuel source, generating technology, online year, and retirement year if any) from the Energy Information Administration (EIA) Form 860.[4] At the time of the data collection (in late 2019/early 2020), the latest complete year of data was the 2018 EIA Form 860. We include generators from this form that are flagged with any of these statuses: operating, standby/backup, new unit under construction, cold standby, out of service but will be returned to service, construction complete but not yet in commercial operation, not under construction but site prep underway, and under construction. Generators are assigned to load zones based on their latitude and longitude.

The EIA Form 860 also includes planned retirement years for generators, if known. Generators are not allowed to operate beyond their expected lifetime, and therefore planned retirement years are used to calculate the lifetime of the plant to be included in the SWITCH runs. Monthly data was used from more recent Form 860 data to confirm that there were no additional planned retirements. For generators with no known retirement years, we assign a maximum lifetime based on the generating technology (Table S3.

For thermal generators, we join the list of generators from Form 860 with the monthly generation and fuel use from the EIA Form 923, for the available years 2004 – 2018.[5] We use the historical monthly generation and fuel use from Form 923 to calculate the second-best (or second-lowest) heat rate (MMBtu/MWh) for each generator, avoiding any outliers from the best heat rate. The heat rate is used in the SWITCH dispatch optimization to calculate the fuel use and associated variable cost and emissions from dispatching thermal generators.

Hydropower generators are constrained to generate at their average historical monthly capacity factor[2]. We extract the monthly generation by generator from 2004 – 2018 (the most recent complete data at the time of the analysis) from the Form 923. For each generator, we calculate the average power for each month (monthly generation/hours in month) and estimate that the proxy for minimum flows for each generator is the power generated at half the average monthly level. Monthly hydropower capacity factors

by generator for the Canadian load zones (British Columbia and Alberta) are used from the prior SWITCH database, based on data from Statistics Canada Tables.[3]

The existing generator set for the Canadian and Mexican load zones are used from the data previously compiled for prior SWITCH-WECC analysis, originally from the WECC Transmission Expansion Planning Policy Committee database of generators, and were not updated for this study.[3,6]

Candidate Generators

One of the key decision variables in SWITCH is the capacity investment of generation, out of a set of candidate generators with specific generating technologies and fuel sources, load zone locations, and other physical and financial generating characteristics. We use the dataset of candidate generators that was previously compiled in prior SWITCH-WECC analyses.[2,3,6]

Candidate onshore and offshore wind generators were derived based on wind power output from a gridded 3TIER Western Wind and Solar Integration Study dataset[8] and a gridded Canadian wind developer dataset, and a selection of prime sites based on criteria including high wind energy density, and proximity to transmission.[3] A portion of candidate generators were screened out in California if they were in “Category 3, high environmental risk” locations, which include areas legally excluded for development, protected areas with ecological or social value, conservation regions, and prime agricultural land.[7]

Candidate solar generators include Residential PV (rooftop PV on homes), Commercial PV (rooftop PV on commercial buildings), Central PV (utility-scale), and Concentrating Solar Power with and without storage (solar thermal trough systems with or without thermal energy storage). Distributed Residential and Commercial PV candidate generation had been derived based on a gridded population density dataset, solar insolation data from NREL’s (now deprecated) Solar Prospector tool, and assumptions on rooftop area and solar cell characteristics.[3] Available land and capacity for Central PV and Concentrating Solar Power candidate generators were screened based on land exclusion criteria (including national parks, wildlife areas, and steep terrain), solar insolation from the System Advisor Model from the National Renewable Energy Laboratory,[8] and assumptions on the solar technology characteristics.[3]

To simulate the dispatch of wind and solar generators, we use an exogenous dataset of hourly capacity factors by generator that had been constructed in prior SWITCH analyses.[2,3] For wind generators, hourly capacity factors for the candidate generator set were calculated from the 3TIER Western Wind and Solar Integration Study wind speed dataset[9] using idealized turbine power curves. For solar generators, hourly capacity factors for the candidate generator set are calculated from the System Advisor, using data from 2006 (consistent with the base weather year underlying the load profiles).[8] Central PV and onshore wind generators with capacity-weighted average capacity factors below the 75th percentile for their technology were screened out to only have the candidate set among a computationally tractable, and commercially viable, set of higher-quality resource sites.[3] For existing solar and wind generators, we average the hourly capacity factors for all solar and wind generators, respectively, in each load zone, and assign all the generators in that load zone the average capacity factor for the given technology.

Biogas (from landfill, wastewater treatment plants, and manure) candidate generator availability is derived from an assessment of the technical resource/feed stock availability.[10] Bioliquid generators are allowed to be reinstalled in their current locations but no new bioliquid plants are assumed. No new biomass (bio solid) candidate generators are assumed, but cogeneration bio solid generation is allowed to be reinstalled at the end of its lifetime.[3] Candidate geothermal generators are based on the current locations and capacity of existing plants that may be reinstalled after retirement.[3] We assume there is no candidate hydropower generation.

Natural gas combined cycle generators and combustion turbines do not have imposed maximum capacity limits. Coal generators are not allowed to be installed in California but are otherwise allowed to expand without capacity limits. Cogeneration plants (with gas combined cycle or combustion turbine plants) are

given the option to be reinstalled at current locations after reaching their maximum age, at current capacity limits. We assume that there is no nuclear generation available for new, candidate generation.

Table S2 summarizes the maximum capacity available for the set of candidate generators, and the capacity installed for existing generators.

Technology assumptions on lifetime, capacity factors, efficiencies

For all existing generators and candidate generators, we assume the following default technology assumptions based on the generating technology and fuel source (Supplementary Table 4), primarily based on the technology and cost assumptions from Black & Veatch[11] and a CEC Cost of Generation Report collected in the prior SWITCH analyses[3,6] and converted to $2018, with some technology values (natural gas combined cycle, natural gas turbine, battery, wind, solar PV, solar CSP, and geothermal) updated based on NREL's 2020 Annual Technology Baseline.[12] For existing generators with known planned retirement years, a specific lifetime is calculated.

Transmission

The SWITCH optimization includes the construction of new transmission lines and the operations of existing and new transmission as decision variables. The model includes a set of 126 existing aggregated transmission lines between load areas within the WECC, based on a prior SWITCH analysis that aggregated the thermal limits of individual high-voltage lines between load areas from a Ventyx purchased dataset and Federal Energy Regulatory Commission (FERC) data.[3] New transmission capacity may be added to existing transmission corridors or constructed between 21 adjacent load zone pairs where there is currently no transmission. The line length assumed is 1.3 times the straight-line distance between the largest substations in each load zone, based on a prior analysis that calculated this as the average ratio between line length and straight-line distance between transmission substations in WECC.[2] For every 100 miles of distance, a 1% efficiency loss is assumed, based on typical losses for high-voltage transmission.[13] For both existing and newly constructed transmission lines, the maximum power transfer on each line is the thermal limit multiplied by a derating factor. The derating factor is from a prior SWITCH analysis and is meant to capture the combined effect of stability concerns, loop flows, voltage concerns, power factors less than unity, and overloading of individual transmission lines within the bundle, that are difficult to model in detail in the linear model. The factors are 0.59 for alternating current (AC) lines, and 0.91 for direct current (DC) lines, of which there are only two in our analysis.[3]

Overnight capital cost, variable O&M cost, fixed O&M cost

The SWITCH optimization includes several types of costs in the decision to invest and/or operate in generation. Costs for candidate generators include overnight capital cost (applied to the built capacity), fixed O&M costs per MW-year of operations, and variable O&M costs per MWh of operation. Mature technologies (biogas, bioliquid, biosolid, coal, gas cogeneration, gas steam turbine) are assumed to have their real costs stay constant over time, whereas other technologies are assumed to decrease costs over time with technology improvements and economies of scale. Capital, fixed O&M, and variable O&M costs by generator technology type originate primarily from Black & Veatch estimates for the mature technologies.[3,11] For technologies with changing costs over time (solar PV, solar CSP, wind, geothermal, gas CCGT, gas CT), we compile cost data from NREL's 2020 Annual Technology Baseline database using the "Moderate Scenario" which is based on the of median projections from the literature.[12] The average costs by energy source, and technology are in Supplementary Table 5. Capital costs for existing generators are considered sunk costs and we do not include them in the total system costs; they do not affect future investment decisions. Variable O&M costs for existing generators are set to be the same as for candidate generators.

Connection costs

In addition to capital costs for the construction of a generator itself, for candidate generators we also add a connection cost to reflect the expense of connecting to the grid. This connection cost originally derived from EIA data and compiled in a prior SWITCH analysis[3] is either a "generic" cost if the generator is not located at a specific site (for gas, coal, biosolid, biogas, or battery storage), or is calculated for a specific

site (for onshore wind, offshore wind, geothermal, central PV, and CSP with and without storage). The "generic" cost includes the cost of building a substation ($80,000/MW in $2018) and a small transmission line to the substation ($31,000 in $2018). Site specific connection costs include a transmission line cost of $1,200 per MW per km based on the distance from the site to the substation, and the cost of the substation. The cost for underwater cable for offshore wind is $6000 per MW per km. There is no connection cost applied for existing generation.

Fuel costs and Emissions

Bio liquid costs ($0.01 / MMBtu) and uranium costs ($1.53 / MMBtu) originate from the EIA's Annual Energy Outlook (AEO) compiled from a prior WECC analysis.[6] [3]These are average costs across all the load zones, in $2018. The fuel costs for bio solid generators are based on supply curves derived based on estimates of the economically feasible volumes of biomass feedstock available by load zone and different fuel price tiers.[3]

Transmission costs

The cost of building new transmission lines is calculated as the product of a line length, a base $/MW/km cost, a terrain multiplier that reflects the topography differences that make a line more expensive to construct, and an economic multiplier that represents differences in labor, permitting, and other "soft" costs between WECC load zones.

We assume a base $960/MW-km cost, which is the cost for constructing a 500 kV line in the WECC from the ReEDS capacity expansion model database ($1,347/MW-mile in $2010 dollars, converted to $/MW-km and $2018 dollars).[13] For the economic multiplier, for all lines within California we use a factor of 2.25, between California and other WECC load zones we use a factor of 1.125, and within other WECC load zones we use a factor of 1 (keeping the base cost as it is) from the the ReEDS documentation.[13] Steeper terrain and urban land area increase transmission construction costs. Terrain multipliers come from a prior SWITCH GIS analysis that overlaid the transmission line paths over a gridded dataset of terrain-dependent transmission costs, that had been derived from the slope and the land cover in the WECC region.[3] The multipliers range from 0.7 to 3.4. Transmission lines also have a fixed O&M cost applied to reflect upkeep costs for lines, which is assumed to be 3% of capital costs from the prior SWITCH analysis.

Load

The future load assumed in this analysis was developed in a prior study and represents a case of high energy efficiency and building electrification, as well as increased adoption of Zero Emissions Vehicles (ZEVs), primarily from electric vehicles.[6] The load forecast achieves a doubling of the rate of energy efficiency by 2030 in California, compliant with the state's SB 350 legislative targets, aggressive building electrification starting in 2020, growing industry electrification, and approximately 125,000 GWh in electricity demand from transportation. Hourly demand profiles from 2006 (consistent with the weather-year used for calculating solar and wind capacity factors) from FERC Form 714 and a dataset procured from ITRON were used as a base from which demand projects (residential, commercial, industrial, transportation) were created and scaled by sector to meet states' policy targets and reflect population growth.[17] Where detailed state/province-level load forecasts with state efficiency, electrification, and population estimates were available (including California, Washington, Oregon, British Columbia, and Alberta) load zone forecasts were scaled to those projections; otherwise forecasts were scaled to the EIA's Annual Energy Outlook projections in the prior SWITCH analysis.[6,14] In the 2017 Annual Energy Outlook Electric projections used, population growth across the U.S. is on average 0.6% annually, based on the U.S. Census Bureau's mid-case projections at the time.[14] Electric vehicles are assumed to charge in an "unmanaged" way (without smart charging or time-of-use rates), based on charging profiles developed with an agent-based mobility model BEAM.[15,16]

Carbon cap

A constraint on carbon emissions from generators can be imposed in SWITCH by load zone and investment period. In this analysis, we force carbon emissions to be zero which effectively disallows the

use of the following fuel sources: coal, distillate fuel oil, gas, residual fuel oil. All other SWITCH fuel sources (i.e. biogas, bio liquid, bio solid and uranium) are unaffected by the carbon cap.[17]

Supplemental References

1. Johnston, J., Henriquez-Auba, R., Maluenda, B. & Fripp, M. Switch 2.0: A modern platform for planning high-renewable power systems. *SoftwareX* **10**, (2019).

2. Nelson, J. *et al.* High-resolution modeling of the western North American power system demonstrates low-cost and low-carbon futures. *Energy Policy* **43**, 436–447 (2012).

3. Mileva, A., Johnston, J., Nelson, J. H. & Kammen, D. M. Power system balancing for deep decarbonization of the electricity sector. *Appl Energy* **162**, 1001–1009 (2016).

4. Form EIA-860 detailed data with previous form data (EIA-860A/860B). https://www.eia.gov/electricity/data/eia860/.

5. Form EIA-923 detailed data with previous form data (EIA-906/920). https://www.eia.gov/electricity/data/eia923/.

6. Wei, M., Raghavan, S. V. & Hidalgo-Gonzalez, P. *Building a Healthier and More Robust Future: 2050 Low-Carbon Energy Scenarios for California*. (2019).

7. Wu, G. C., Torn, M. S. & Williams, J. H. Incorporating land-use requirements and environmental constraints in low-carbon electricity planning for California. *Environ Sci Technol* **49**, 2013–2021 (2015).

8. Blair, N. *et al.* System Advisor Model (SAM) General Description (Version 2017.9.5). (2018).

9. National Renewable Energy Laboratory. Western Wind Data Set. Preprint at https://www.nrel.gov/grid/western-wind-data.html.

10. Milbrandt, A. A Geographic Perspective on the Current Biomass Resource Availability in the United States. (2005).

11. Black & Veatch. *Cost and Performance Data for Power Generation Technologies, Prepared for the National Renewable Energy Laboratory*. https://refman.energytransitionmodel.com/publications/1921 (2011).

12. NREL (National Renewable Energy Laboratory). 2020 Annual Technology Baseline. Preprint at https://atb.nrel.gov/ (2020).

13. Brown, M. *et al.* Regional Energy Deployment System (ReEDS) Model Documentation: Version 2019. (2020).

14. Energy Information Administration. Assumptions to the Annual Energy Outlook 2017. (2017).

15. Sheppard, C., Waraich, R., Campbell, A., Pozdnukhov, A. & Gopal, A. R. Modeling plug-in electric vehicle charging demand with BEAM The framework for behavior energy autonomy mobility. (2017).

16. Szinai, J. K., Sheppard, C. J. R., Abhyankar, N. & Gopal, A. R. Reduced grid operating costs and renewable energy curtailment with electric vehicle charge management. *Energy Policy* **136**, 111051 (2020).

17. U.S. Energy Information Administration. Carbon Dioxide Emissions Coefficients. https://www.eia.gov/environment/emissions/co2_vol_mass.php (2021).
